# New genetic prognostic biomarkers in primary central nervous system lymphoma (PCNSL)

**DOI:** 10.1002/brb3.2061

**Published:** 2021-02-16

**Authors:** Diego Gomes Candido Reis, Débora Levy, Luís Alberto de Pádua Covas Lage, Hebert Fabrício Culler, Vanderson Rocha, Sérgio Paulo Bydlowski, Maria Cláudia Nogueira Zerbini, Juliana Pereira

**Affiliations:** ^1^ Department of Hematology, Hemotherapy and Cell Therapy Laboratory of Medical Investigation in Pathogenesis and Directed Therapy in Onco‐Immuno‐Hematology (LIM‐31) Faculdade de Medicina da Universidade de São Paulo (FMUSP) São Paulo SP Brazil; ^2^ Laboratory of Immunology (LIM19) Heart Institute (InCor) Faculdade de Medicina Hospital das Clínicas HCFMUSP Universidade de São Paulo São Paulo SP Brazil; ^3^ Instituto do Câncer do Estado de São Paulo (ICESP) São Paulo Brazil; ^4^ Fundação Pró‐Sangue Hemocentro de São Paulo São Paulo Brazil; ^5^ Churchill Hospital Oxford University Oxford UK; ^6^ Department of Pathology Faculdade de Medicina da Universidade de São Paulo (FMUSP) São Paulo SP Brazil

**Keywords:** biomarkers, central nervous system neoplasms, gene expression, lymphoma, survival analysis

## Abstract

**Background:**

PCNSL is a rare extranodal NHL with poor prognosis. Tumorigenesis has been associated with hyperactivation of BCR downstream and NFkB pathways. We studied the prognosis of the relative expression profile of target genes of NFkB pathway (*MYC, BCL2)*, the essential transcriptional regulator in hematopoiesis *LMO2,* the checkpoint regulation pathway *MGMT*, the transcription factor *POU2F1,* the immune checkpoint gene *PDCD1,* and the proto‐oncogene and transcriptional repressor gene *BCL6* and its proteins in PCNSL.

**Methods:**

This study is a retrospective cohort study; 35 immunocompetent PCNSL‐DLBCL patients had their gene expression (RT‐qPCR) normalized to internal control gene *GUSB*.

**Results:**

Median patient age was 62 years, median OS was 42.6 months (95% CI: 26.6–58.6), PFS was 41 months (95% CI: 19.7–62.4), and DFS was 59.2 months (95% CI 31.9–86.6). A moderate correlation was found between the gene/protein expressions of MYC (kappa = 0.596, *p* = .022) and of BCL2 (kappa = 0.426, *p* = .042). Relative gene expression of *MYC* ≥ 0.201 (HR 6.117; *p* = .003) was associated with worse 5‐year OS. Relative gene expression of *MYC* ≥ 0.201 (HR 3.96; *p* = .016) and *MGMT* ≥ 0.335 (HR 3.749; *p* = .056) was associated with worse PFS. Age > 60 years and IELSG score moderate/high were also associated with worse prognosis.

**Conclusions:**

Overexpression of *MYC* and overexpression of *MGMT* were prognostic markers associated with unfavorable clinical outcomes in PCNSL.

## Graphical abstract

In this study, we evaluated the expression of target genes of NFkB pathway (MYC, BCL2), the essential transcriptional regulator in hematopoiesis (LMO2), the checkpoint regulation pathway MGMT, the transcription factor POU2F1, and the immune checkpoint gene PDCD1 in patients with PCNSL.

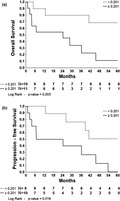

## 1 INTRODUCTION

Primary central nervous system lymphoma (PCNSL) is an aggressive extranodal non‐Hodgkin lymphoma restricted to the brain, meninges, eyes, and spinal cord (Louis et al., 2016; Swerdlow et al., 2017). Despite its rarity (incidence of 0.7/100.000 persons per year), the rate has been increasing by 1.6% per year in the last decade, mainly in elderly people (Shiels et al., 2016).

It is described that enrichment of mutations involving the B‐cell receptor signaling pathway and hyperactivation of the NF‐kappaB prosurvival signaling pathway (NFkB) are associated with the pathogenesis of PCNSL; however, the rarity of this disease and the paucity of histopathological material available area challenge for molecular studies (Braggio et al., 2015; Rubenstein, 2017).

Some studies have suggested the gene expression of *MYC, BCL2, LMO2, MGMT, POU2F1, PDCD1,* and *BCL6* as prognostic biomarkers in PCNLS. *MYC* is a proto‐oncogene, and its protein is a nuclear transcription factor involved in cell cycle progression and apoptosis, being associated with worse prognosis in patients with systemic DLBCL (Kawamoto et al., 2016; Perry et al., 2014). Its expression is upregulated in PCNSL, which may lead to lymphomagenesis (Fischer et al., 2011; Grommes et al., 2017). Copy number alterations and translocations at chromosome 9p24 have been described, involving the genes coding for programmed death ligand 1 (PD‐L1) and PD‐L2, suggesting that immune escape may also be involved in the pathogenesis of PCNSL, with potential benefit of programmed cell death 1 (PD‐1) blockade therapy (Chapuy et al., 2016). MGMT, a tumor suppressor gene that has been associated with PCNSL (Deckert et al., 2014; Deckert, Montesinos‐Rongen, et al., 2014), codifies a protein that repairs DNA, and may be silenced by methylation of the promotor region. Overexpression of MGMT is associated with worse prognosis, and methylation of its promotor region was associated with better overall survival among patients treated with high‐dose chemotherapy, and with a better tumor response in patients treated with temozolomide (Kurzwelly et al., 2010).

PCNSL often presents translocation involving immunoglobulin (Ig)‐related genes, including BCL6 (Basso & Dalla‐Favera, 2012), leading to constitutive activity and to tumorigenesis (Cattoretti et al., 2005). BCL2 is a proto‐oncogene regulating cell cycle and apoptosis, and its expression is associated with lymphomagenesis (Vaux et al., 1988) and is a biomarker of worse prognosis in DLBCL (Kawamoto et al., 2016; Perry et al., 2014). LMO2 forms a transcriptional complex that regulates gene expression in DLBCL and is usually overregulated in NHL and is associated with improved prognosis in both DLBCL and PCNSL, being overexpressed in 52% of PCNSL (Cubedo et al., 2012; Lossos et al., 2014). POU2F1 is usually overexpressed in NHL, which may lead to higher susceptibility to therapies as irinotecan, paclitaxel, and mutations on POU2F1 may lead to tumor resistance against chemotherapies (Gupta et al., 2012).

The increasing incidence of PCNSL, mainly in elderly population, who frequently presents comorbidities and are more vulnerable to oncological treatments (Abrey et al., 1998; Kasenda et al., 2015), the aggressiveness of this disease, the current toxicity profile of the therapies, and the heterogeneity in patient response to therapy and outcomes (Abrey et al., 2005) demand further research on biomarker identification that could help to identify potential targets for new therapies and provide prognostic information.

## 2 METHOD

### 2.1 Study population

The Local Institutional Review Board in accordance with the Declaration of Helsinki approved this study (IRB No. 1.266.854, approval date: 7 October 2015). All immunocompetent patients consecutively diagnosed with PCSNL at a reference center for cancer in Brazil (University of São Paulo) between January 1995 and December 2016 were retrieved. Inclusion criteria consisted of age ≥ 18 years and histologic confirmation of PCNSL histopathology DLBCL CD20+. Exclusion criteria consisted of patients with immunodeficiency, systemic lymphoma, and unavailable medical records/paraffin blocks.

Baseline clinical, laboratory, and disease features were retrieved from medical records. Cranial magnetic resonance imaging (MRI), bone marrow biopsy, and neck, chest, abdomen, and pelvis computerized tomography (CT) data were also collected. International Extranodal Lymphoma Study Group Prognostic Score (IELSG) was calculated as described elsewhere (Ferreri et al., 2003).

Patients were treated with high‐dose methotrexate (HDMTX 3.5–5 g/m^2^ IV, five cycles each 15 days, 3 hr infusion) combined or not with other chemotherapeutical agents as induction treatment. Consolidation therapy was prescribed for patients who achieved complete response (CR) after induction therapy, consisting of whole‐brain radiotherapy (WBRT) or up to 2 cycles of cytarabine (2 g/m^2^ IV, on D1 and D2). Patients with poor clinical conditions were treated with WBRT (30–40Gy, up to 20 fractions) or best support care. Tumor response was evaluated according to the International PCNSL Collaborative Group (IPCG), and active follow‐up was performed (Abrey et al., 2005).

### 2.2 Immunohistochemistry

Two hematopathology experts reviewed the tumor histology according to the WHO criteria (Swerdlow et al., 2017). Four‐millimeter sections from formalin‐fixed paraffin‐embedded (FFPE) tumor samples obtained at diagnosis were stained with hematoxylin–eosin (HE), as previous described (Grizzle el al., 1998), followed by an initial immunohistochemistry (IHC) panel with monoclonal antibodies (MoAbs) CD3 and CD20. If the initial analysis was suggestive of PCNSL histopathology diffuse large B‐cell lymphoma, the IHC panel was expanded (Swerdlow et al., 2017).

Briefly, antigen retrieval was performed using citrate buffer in a Pascal pressure cooker set to a temperature of 125°C and a pressure of 18 psi (Celerus RIPTIDE; Celerus Diagnostics). Endogenous peroxidase was blocked, and the samples were incubated overnight in a solution containing monoclonal antibodies for MYC (clone Y69; Abcam) at a dilution of 1:100 in pH 9.0, BCL2 (clone 124, Cell Marque) at a dilution of 1:100 in pH 9.0, LMO2 (clone CP51; Cell Marque) at a dilution of 1:50 in pH 9.0, MGMT (clone MT3.1; Abcam) at a dilution of 1:400 in pH 9.0, POU2F1 (clone AB151; Abcam) at a dilution of 1:200 in pH 9.0, and PDCD1 (clone NAT 105; Abcam, USA) at a dilution of 1:100 at pH 9.0. Samples were incubated with Novolink Polymer (Leica Biosystems Newcastle) for 30 min and then incubated in the chromogenic substrate diaminobenzidine (DAB, from DBS) and counterstained with hematoxylin. Slides were mounted using a synthetic resin (Entellan; Merck). Due to the lack of enough histopathological material, MoAb for BCL6 was not performed. The positive score was determined as the percentage of positive tumor cells using a semiquantitative method in 25% increments under an optical microscope (40X objective). Two distinct observers analyzed the slides.

### 2.3 Molecular biology

Total RNA was extracted from FFPE tissues (5‐μm‐thick) using the Qiagen FFPE RNeasy Kit (Valencia) with a modified deparaffinization step (Belder et al., 2016). Briefly, sections were deparaffinized by two repeated incubations in xylene for 10 min, followed by two repeated incubations in 100% ethanol for 5 min, and then washed with distilled water for 30 s. After deparaffinization, the remaining steps of RNA extraction were followed according to the Qiagen FFPE RNeasy Kit manual. All RNA samples were analyzed spectrophotometrically on a NanoDrop (NanoDrop 1,000 Spectrophotometer V3.7; Thermo Fisher Scientific, Wilmington, DE, USA). cDNA was synthesized from 1.0 µg of total RNA from 35 FFPE tumor samples and 5 normal lymph node samples (normal control samples) using the High Capacity cDNA Reverse Transcription Kit (Applied Biosystems) according to the manufacturer's protocol. The integrity of the cDNA template was evaluated using the endogenous housekeeping gene GUSB (Catalog number: 4310888E; Applied Biosystems, Foster City, CA, USA). Subsequently, qRT‐PCR was carried out for *MYC* (Hs 202453), *BCL2* (Hs 0060823), *LMO2* (Hs 00277106), *MGMT* (Hs 501522), *POU2F1* (Hs 283402), *PDCD1* (Hs 158297), and *BCL6* (Hs 478588).

Standard curves were prepared using total RNA from a pool of five normal lymph nodes. The 5 × 10‐fold serial dilutions were prepared from the original cDNA stock and TaqMan qRT‐PCR assays performed on a 7,500 FAST Real‐Time PCR System (Applied Biosystems) using Life Technologies Applied Biosystems TaqMan Gene Expression Assays according to the manufacturer's instructions and TaqMan® Gene Expression PCR Master Mix (Applied Biosystems). Expression ratios were calculated based on relative quantification. Samples with *GUSB* Ct values > 34 were not included. The sample acceptance criterion was based on the Minimum Information for Publication of Quantitative Real‐Time PCR Experiment (MIQE) guidelines (Bustin et al., 2009) and the determined PCR efficiency for each assay using FFPE for GUSB, and all tested genes (all 94.1%–103.5%).

### 2.4 Statistical analysis

Descriptive statistics were calculated to summarize clinical and molecular characteristics. Gene expression and protein expression by IHC were dichotomized into low and high expression levels using the receiver operating characteristic (ROC) analysis (Choi, 1998). Optimal cutoff points for relative gene expression and immunohistochemistry were determined based on the best balance of sensitivity and specificity along with larger increases in LRs, which best differentiate clinical outcomes. The univariate analysis to assess the association among categorical variables was performed using the Fisher exact test. A Cox univariate analysis was performed to estimate the association between categorical variables and survival curves (Fischer, 1922). Due to small sample, multivariate analysis was not performed.

Overall Survival (OS), progression‐free survival (PFS), and disease‐free survival (DFS) were determined using the Kaplan–Meier method. The log‐rank test was used to compare survival curves and to verify the association between categorical variables and survival curves. A correlation test between gene expression and IHC was performed, using the Youden index and Kappa coefficient (Youden, 1950). All analyses were performed using Statistical Package for the Social Sciences (SPSS) version 11.0. *p* values ≤ .05 were considered statistically significant.

## 3 RESULTS

Fifty‐one patients were initially screened and retrospectively evaluated, and 35 patients (68.6%) were enrolled; 16 patients were excluded due to unavailability of paraffin block, medical records, or after histology revision. Patient's characteristics are shown in Table 1. Median follow‐up for all patients was 41 months (range, 1–172). Twenty‐six patients (74.2%) were dead at the cutoff date, with a median interval between diagnosis and death of 26.6 months (range, 1–92). The main cause of death described was progression disease (PD) in 21 patients (80.7%).

**Table 1:** Characteristics of all PCNSL patients

Characteristics	Total (*N* = 35)	Included in gene analysis (*N* = 21)	Comparison between total population and subgroup *p* value
Median age (range)	62 (26–84)	68 (26–84)	.493
> 60 years	19 (54.3)	14 (66.7)	.412
Gender			
Female	20 (57.1%)	13 (61.9%)	.785*
Male	15 (42.9%)	8 (38.1%)	
IELSG prognostic score			
Low risk (0 + 1)	7 (20%)	2 (9.5%)	.572*
Intermediate risk (2 + 3)	17 (48.6%)	10 (47.6%)	
High risk (4 + 5)	11 (31.4%)	9 (42.9%)	
ECOG			
≤2	20 (57.1%)	10 (47.6%)	.584*
>2	15 (42.9%)	11 (52.4)	
Deep CNS involvement ^a^			
Yes	27 (77.1%)	18 (85.7%)	.508*
No	8 (22.9%)	3 (14.3%)	
Treatment based on HDMTX^b^			
No	5 (15.2%)	3 (15.8%)	1*
Yes	28 (84.8%)	16 (84.2%)	
Response type after 1st line ^c^			
CR	15 (45.5%)	7 (36.8%)	.715*
PR	9 (27.3%)	7 (36.8%)	
Progression disease	9 (27.3%)	5 (26.3%)	
WBRT ^d^, ^e^			
No	14 (40%)	7 (33.4%)	.777*
Yes	21 (60%)	14 (66.6%)	
Relapse after 1st line ^f^			
No	12 (80%)	5 (71.4%)	1*
Yes	3 (20%)	2 (28.6%)	
Refractory disease ^g^			
No	24 (72.7%)	14 (73.7%)	1*
Yes	9 (27.3%)	5 (26.3%)	
Death cause ^h^			
Progression disease	21 (80.7%)	12 (80%)	1*
Other	5 (19.3%)	3 (20%)	

Note

Data are presented as *n* (%) or median (range).

Abbreviations: CNS, central nervous system; CR, complete response; ECOG, Eastern Cooperative Oncology Group; HDMTX, high‐dose methotrexate induction chemotherapy; IELSG, International Extranodal Lymphoma Study Group; Modified IPI, Modified International Prognostic Index; PR, partial response; WBRT, whole‐brain radiotherapy.

^a^ Deep nervous system involvement is characterized by periventricular regions, basal ganglia, brainstem, and/or cerebellum.

^b^ Percentage calculated among patients who received at least 1 line of therapy and who were treated with HDMTX.

^c^ Percentage calculated among patients who received at least 1 line of therapy.

^d^ WBRT 40 grays in 20 fractions was prescribed either as consolidation treatment or as monotherapy for patients who did not tolerate chemotherapy or after progression.

^e^ Percentage calculated among patients treated with WBRT.

^f^ Percentage calculated among patients who achieved CR after 1st line.

^g^ Percentage calculated among patients who received at least 1 line of therapy.

^h^ Percentage calculated among patients who died.

* Student's *t* test; **Independent *t* test.

**Figure 1:**
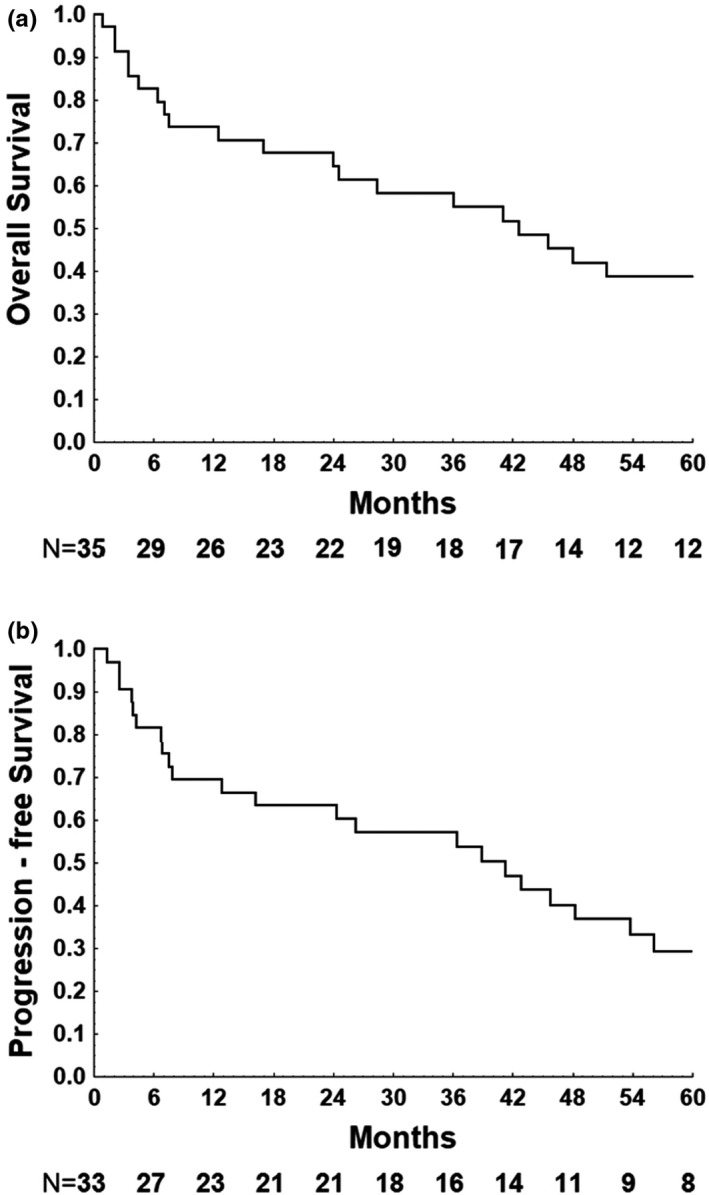
Kaplan–Meier survival curve for 5‐year overall and progression‐free survival of 35 and 33 patients with PCNSL, respectively. (a): Overall survival. (b): Progression‐free survival

The median OS was 42.6 months (95% CI: 26.6–58.6). The OS rates at 24, 48, and 60 months were 65.5%, 43.8%, and 40.4%, respectively. The median PFS was 41 months (95% CI: 19.7–62.4), and the median DFS was 59.2 months (95% CI 31.9–86.6). Figure 1 shows survival curves. Age higher than 60 years, IELSG score ≥ 2 (moderate/high), ECOG ≥ 3, treatment without WBRT, and refractory disease were statistically significant predictors of worse survival in univariate analysis (Table 2).

**Table 2:** Main results of univariate analysis

Characteristic	5‐year overall survival		Progression‐free survival		Disease‐free survival	
	HR (CI 95%)	*p* value	HR (CI 95%)	*p*‐value	HR (CI 95%)	*p* value
Age(> 60 y/o versus ≤ 60 y/o)	2.466 (1.09–5.57)	.025	2.857 (1.23–6.58)	.01	3.333 (0.77–14.37)	.088
IELSG score (≥2 versus < 2)	3.564 (1.05–12.03)	.029	4.151 (1.19–14.44)	.001	0.177 (0.02–1.48)	.076
ECOG (≥ 3 versus < 2)	7.005 (2.67–18.37)	<.001	3.951 (1.65–9.45)	.001	10.272 (1.80–58.39)	.002
WBRT (Yes versus No)	3.413 (1.51;7.68)	<.001	2.281 (1.02;5.06)	.037	4.126 (0.96;17.62)	.039
Refractory disease (yes versus no)	28.272 (5.82–137.27)	<.001	75.672 (8.96–638.73)	<.001	^c^	
Anti‐MYC (>50% versus ≤ 50%)	1.186 (0.50–2.80)	.696	1.519 (0.60–3.85)	.374	1.392 (0.27;6.93)	.687
Anti‐BCL2 (>50% versus ≤ 50%)	3.559 (0.83–15.19)	.067	3.376 (0.78–14.53)	.083	^d^	
Anti‐LMO2 (≤25% versus > 25%)	1.67 (0.73–3.79)	.215	1.677 (0.71–3.93)	.229	^d^	
Anti‐MGMT (positive versus negative)	^a^		^a^		^a^	
Anti‐PDCD1 (positive versus negative)	0.516 (0.12–2.215)	.364	0.838 (0.19–3.58)	.811	^d^	
Anti‐POU2F1 (positive versus negative)	^b^		^b^		^b^	
*MYC* (≥0.201041 versus < 0.201041)	6.117 (1.61–23.14)	.003	3.96 (1.19–13.14)	.016	2.924 (0.26–32.93)	.364
*BCL2* (≥0.3516 versus < 0.3516 )	1.934 (0.65–5.73)	.227	1.98 (0.65–6.02)	.221	1.423 (0.12–16.04)	.774
*LMO2* (<0.689232 versus ≥ 0.689232)	2.099 (0.74–5.89)	.151	2.718 (0.87–8.49)	.073	0.237 (0.02–2.67)	.207
*MGMT* (≥0.335666 versus < 0.335666 )	1.793 (0.54–5.89)	.331	3.749 (0.87–15.99)	.056	^d^	
*POU2F1* (<1.44819 versus ≥ 1.44819)	1.179 (0.45–3.06)	.735	0.909 (0.33–2.47)	.852	1.732 (0.23–12.78)	.586
*PDCD1* (<2.00938 versus ≥ 2.00938)	1.539 (0.34–6.96)	.573	1.762 (0.38–7.97)	.456	^d^	
*BCL6* (<2.73477 versus ≥ 2.73477 )	0.952 (0.35–2.54)	.922	0.815 (0.30–2.21)	.687	4.472 (0.27–71.80)	.247

^a^ Could not be calculated, because all patients had no expression of anti‐MGMT.

^b^ Could not be calculated, because all patients presented anti‐POU2F1 > 75%.

^c^ Not calculated since refractory disease is directly related to DFS.

^d^ Since coefficients did not converge, no further models could be fitted.

### 3.1 Gene expression analysis

Gene expression (qRT‐PCR) was performed in all 35 patients; however, gene expression of *MYC, BCL2, LMO2, MGMT, POU2F1, PDCD1,* and *BCL6* was feasible in only 21 (60%), 21 (60%), 21 (60%), 21 (60%), 23 (65.7%), 21 (60%), and 23 (65.7%) patients, respectively. Subjects with absent values of gene expression had the qRT‐PCR repeated and confirmed. Comparison between the variables in this subgroup and the total population did not show any clinically significant difference; therefore, the subgroup was representative of the total population. Table 3 describes the relative gene expression.

**Table 3:** Relative gene expression per patient

Patient	*MYC*	*PDCD1*	*POU2F1*	*MGMT*	*BCL2*	*BCL6*	*LMO2*
P1	0.1103834	0	0	0	0.09072	1.20327293	0.68923207
P2	NA	NA	NA	NA	NA	NA	NA
P3	0	0	4.385562	0	0.13021	4.92333543	2.57602771
P4	0	0	1.567702	0.335666	0.64488	1.70344228	0.15703952
P6	0	0	0	0	0.19728	3.29719402	7.14032912
P7	0.3405111	0	1.72209	0	1.84167	3.27660241	0.13764646
P8	0.2351414	0	0	0	2.2052	0.64762351	0
P9	NA	NA	NA	NA	NA	NA	NA
P10	0.2017973	1.0351888	1.033221	1.417807	0.7658	3.787009	4.07845604
P11	0.2351788	9.2146338	0	0	0.3516	8.3368464	10.9727002
P13	0	0	3.122622	0	0	0	0
P14	0.3040957	0	6.14149	0	0.08092	2.715904	1.6358415
P15	0.412259	0	2.783416	0	0.52765	2.73477377	0.94713051
P16	NA	NA	NA	NA	NA	NA	NA
P17	0.0301969	0	1.448191	0.010951	0.07898	0.63889135	1.99124978
P18	0.371486	0	0	0	1.47173	1.11302554	0.45144126
P19	NA	NA	0	NA	NA	0	NA
P21	NA	NA	0	NA	NA	0	NA
P22	0.4745787	1.729677	1.682568	0	5.56582	7.37556864	17.5060425
P24	NA	NA	NA	NA	NA	NA	NA
P25	NA	NA	NA	NA	NA	NA	NA
P26	0	6.4048083	6.30088	0	1.15876	9.09111926	1.05112574
P27	NA	NA	NA	NA	NA	NA	NA
P28	NA	NA	NA	NA	NA	NA	NA
P30	NA	NA	NA	NA	NA	NA	NA
P31	NA	NA	NA	NA	NA	NA	NA
P32	NA	NA	NA	NA	NA	NA	NA
P36	NA	NA	NA	NA	NA	NA	NA
P37	0.0701626	0	4.481112	0.218243	0.32391	1.63956511	0.06489237
P40	0.214366	0	0	0	0	0.2707965	0.43562553
P41	0.6413885	4.158813	0	0.070006	0.65164	5.446246	7.78468333
P42	0.1977622	2.6626342	2.827798	2.524375	1.18919	3.3456055	13.7798915
P43	0.2010405	2.0093818	2.134024	10.07229	0.56432	3.42655195	6.55950292
P44	0.1775013	0.5917454	0.567903	1.31804	1.19433	1.70547543	3.4625705
P51	NA	NA	NA	NA	NA	NA	NA
Mean	0.20085	1.3241373	137.3782	0.760352	0.90641	2.89908039	3.87721089
Median	0.2010405	0	1.448191	0	0.56432	2.715904	1.6358415

**Figure 2:**
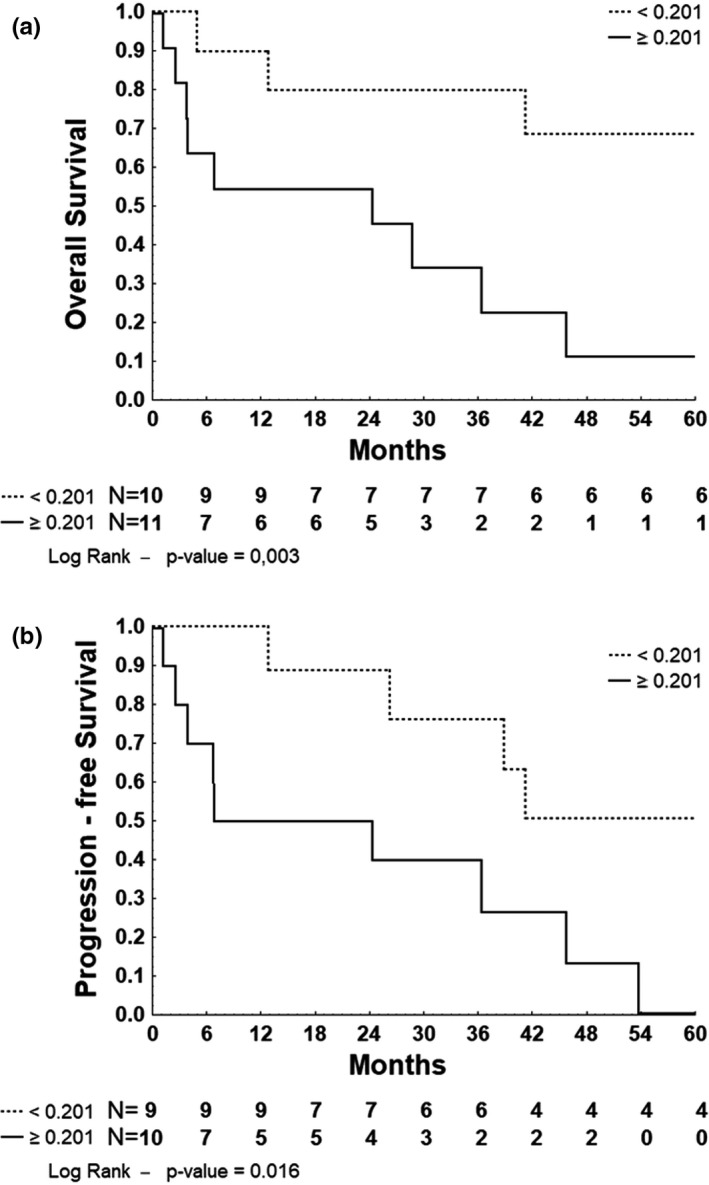
Kaplan–Meier survival curve for 5‐year overall and progression‐free survival of 21 and 19 patients with PCNSL stratified by MYC normalized gene expression, respectively. (a): Overall survival. (b): Progression‐free survival

**Figure 3:**
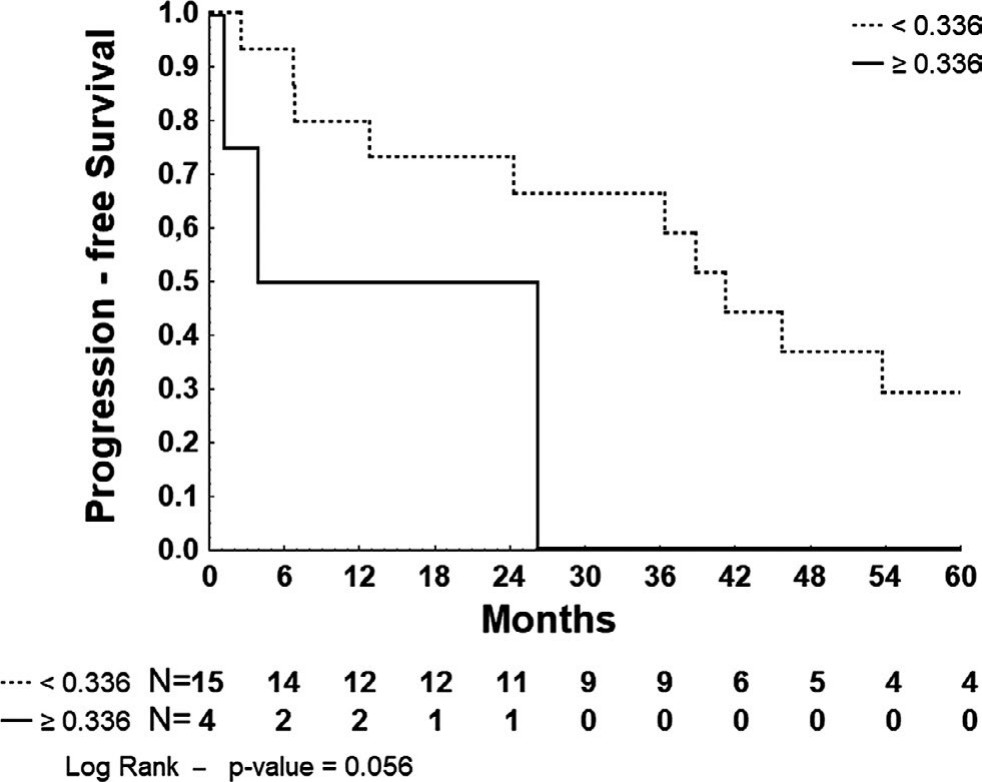
Kaplan–Meier survival curve for 5‐year progression‐free survival of 19 patients with PCNSL stratified by MGMT‐normalized gene expression

Gene expression was analyzed as a qualitative categorical variable taking into account values < or ≥the cutoff obtained by the ROC curves. The cutoff of positivity was 0.201041 for *MYC*, 0.3516 for *BCL2*, 0.689232 for *LMO2*, 0.335666 for *MGMT*, 1.44819 for *POU2F1*, 2.00938 for *PDCD*1, and 2.73477 for BCL6. As described in Table 2, the overexpression of *MYC* was associated with worse OS (HR: 6.117, 95% CI: 1.617–23.149, *p* = .003) and with worse PFS (HR: 3.960, 95% CI: 1.192–13.147, *p* = .016) (Figure 2). Overexpression of *MGMT* was associated with worse PFS (HR: 3.749, 95% CI: 0.879–15.993, *p* = .056) (Figure 3) in univariate analysis.

### 3.2 Immunohistochemistry

Due to the lack of histopathological material, IHC for MYC, BCL2, LMO2, MGMT, POU2F1 and PDCD1 was performed in 33 (94.3%), 32 (91.4%), 32 (91.4%), 31 (88.6%), 33 (94.3%), and 33 (94.3%) patients. Immunohistochemistry for BCL6 was not prospectively performed due to lack of histopathological material. ROC curves using clinical endpoints were performed to establish the cutoff of positivity, which were 50% for MYC, 50% for BCL2, 25% for LMO2, 1% for MGMT, 1% for POU2F1, and 1% for PDCD1.

MYC protein expression > 50% was found in 21 patients (63.6%), BCL2 > 50% in 27 (84.5%), LMO2 > 25% in 18 (59.37%), MGMT > 1% in 0 (0%), POU2F1 > 1% in 33 (100%), and PDCD1 > 1% in 3 (9%). A correlation test was performed between *MYC* gene expression (≥0.201041 versus < 0.201041) and its protein (>50% versus ≤ 50%), and it was classified as moderate (kappa value = 0.596, *p* = .022), with sensitivity of 77.8% and specificity of 81.8%. The correlation test between *BCL2* gene expression (≥0.3516 versus < 0.3516) and its protein (>50% versus ≤ 50%) showed also a moderate correlation (kappa value = 0.426, *p* = .042), with a sensitivity of 97.5% and specificity of 100%. There was a weak correlation between *LMO2* gene expression and its protein (kappa value = 0.243, *p* = .384). Correlation tests between MGMT, POUF2A1 and PDCD1 gene expression and its proteins could not be performed because most patients had gene expression or protein expression above or below the cutoff values.

## 4 DISCUSSION

Despite its small sample, this study is aligned with most molecular studies with PCNSL, which usually are retrospective cohort studies, with populations usually smaller than 50 patients (Braggio et al., 2015; Mrugala et al., 2009; Rubenstein, 2017). Similar to other studies, mean age of diagnosis was 62 years and approximately 30% of them were older than 70 years. The increasing incidence of PCNSL in elderly population has an impact on the choice of treatment and its tolerability (Siegal & Bairey, 2019). In this trial, despite majority of patients had ECOG PS ≥ 2 and age > 60 years, 94.2% received any kind of oncological therapy with curative intent. Ferreri and al. described that 98% of patients with ECOG PS ≥ 2 were able to receive at least 1 line of therapy (Ferreri et al., 2003). Age > 60 years and ECOG PS ≥ 2 were the first poor prognostic biomarkers identified in PCNSL (Nelson et al., 1992; Schultz et al., 1996) and were also associated with worse prognosis in this cohort.

The IELSG score was developed by Ferreri et al., allowing to distinguish patients with different prognosis according to the number of factors. In our study, IELSG moderate/high was associated with poor OS, DFS, and PFS and higher relapse rate, confirming the prognostic value of IELSG score system. Most patients were treated with HDMTX‐based chemotherapy, with response rate (RR) of 72.8% and refractory disease rate (RDR) of 27%, similar to literature data (RR 69% and RDR 30%) (Ferreri et al., 2003). WBRT was prescribed as consolidation regimen or as monotherapy for patients with low clinical status. Currently, there are different options besides WBRT for consolidation therapy in PCNSL, with less neurological toxicity and improved OS and PFS. However, when compared to the best supportive care in frail patients, WBRT may increase overall survival, as shown by Song et al in a retrospective study with 82 patients (Song, 2019). In our study, WBRT was associated with better outcomes (OS, DFS, and PFS); however, due to the small sample, WBRT as consolidation therapy and as monotherapy were analyzed together; therefore, the benefit of WBRT should be interpreted with caution. Also, as the life expectancy of this population increases, bigger attention should be paid to long term toxicities, and WBRT has been associated with a decline in motor functions and learning in 50% of patients up to 2 years after therapy (Citterio et al., 2017), and it was not the focus of this study. Anti‐CD20 therapies were not prescribed for patients in this cohort, neither bone marrow transplantation.

With a median follow‐up of 41 months (range, 1.1–171.8) and median OS of 42.6 months (95% CI 26.6–58.6), the majority of patients were dead at the time of cutoff (26/35, 74%), and at least 80.7% (21/26) had progression disease as the causa mortis. The OS was similar to literature date, ranging from 20.6 months (95% CI 12.4–33.4) in patients treated with WBRT alone to 60 months in patients treated with HDMTX‐based regimen, and the main causa mortis reported in the literature is also PD in 87% of the cases (Abrey et al., 2000; Bessell et al., 2004; DeAngelis et al., 2002; Ferreri et al., 2003).

There was no gene amplification in between 34.3% and 40% of the samples, which may have occurred due to the long‐term storage of the FFPE and the small amount of tissue (Abrahamsen et al., 2003; von Smolinski et al., 2005). Nonetheless, there was no difference statistically significant between the total samples and the amplified samples concerning the variables analyzed. Cutoff points were selected because some samples presented undetermined Ct for the selected genes, with the endogenous reference gene GUSB amplified. This may have occurred because those genes were not expressed or expressed in few cells and in a low level (McCall et al., 2014).

Whole‐exome sequencing has shown NFkB activation in PCNSL, suggesting it as a critical step to tumorigenesis (American Society of Hematology, 2016). *BCL2* regulates programmed cell death, inclusive in lymphocyte, and when overexpressed may block apoptosis. It is associated with chemotherapy and radiotherapy resistances when overexpressed, leading to worse prognosis (Mounier et al., 2003). BCL2 expression not related to t(14;18) (q32;q21) is frequent (Deckert, Brunn, et al., 2014; Deckert, Brunn, et al., 2014). Similar to literature, in this cohort most patients presented overexpression of BCL2 protein (80%), which a tendency for association with poor prognosis (worse OS and PFS; *p* values = .067 and 0.083, respectively) (Cai et al., 2019; Montesinos‐Rongen et al., 2008; Niparuck et al., 2019). In pre‐rituximab era, BCL2 expression was considered a poor prognostic factor in NHL; however, the overexpression of BCL2 in the post‐rituximab era in DLBCL has shown no prognostic value (Mounier et al., 2003). In this trial, no patient was treated with rituximab.

Aligned with other studies, overexpression of *MYC* was associated with poor prognosis (worse OS and PFS; *p* values = .003 and .016, respectively). MYC is a transcriptional factor that regulates diverse array of cellular function, including proliferation, growth, and apoptosis. Its rearrangement and overexpression play important role in the pathogenesis of B‐cell lymphomas, with prognosis impact. Most patients presented overexpression of MYC protein (60%), similar to other studies in which nuclear positivity for MYC was 54% (cutoff value 50%). MYC protein expression was not a prognostic biomarker, similar to data from a prospective trial with 42 patients (MYC protein expression cutoff 50%) (Rubenstein, Hsi, et al., 2013; Rubenstein, Gupta, et al., 2013).

There was a moderate correlation between gene expression and protein expression for MYC and BCL2 (kappa between 0.41 and 0.60). Usually, the transcriptome is not directly proportional to the protein level, being insufficient to explain the correlation between the genotype and its phenotype (Liu et al., 2016). Possible reasons include the lack of protein stability, transcription and translational rate, mRNA degradation, and post‐translational and post‐transcriptional regulations (Bujak et al., 2015; Liu et al., 2016).

In literature, LMO2 and BCL6 protein expression are reported in 52% and 56% of PCNSL, respectively (Lossos et al., 2014). LMO2 is expressed in normal germinal center (GC) B cells and GC‐derived DLBCL, being correlated with better outcome in DLBCL (Alizadeh et al., 2011; Natkunam et al., 2008). Most patients (51.4%) had overexpression of LMO2 in immunohistochemistry, superior to literature data. In a PCNSL study with 32 patients, Four et al showed overexpression of LMO2 in 37% of the patients; however, the cutoff value for positivity was higher (30%) (Four et al., 2017). LMO2 expression is considered a prognostic biomarker not only in hematological malignancies, but also in solid tumors as pancreatic cancer and lung cancer (Aly et al., 2016; Cai et al., 2019; Nakata et al., 2009; Toffolatti et al., 2014). In a retrospective cohort with 49 PCNSL, Lossos et al. (2014) showed that LMO2 protein expression was associated with longer OS. BCL6 is an oncogene that functions as a transcriptional repressor necessary for GC formation (Cattoretti et al., 2005), being expressed 60%‐80% of PCNSL (Montesinos‐Rongen et al., 2008). A substitution in the promotor of the BCL6 gene results in constitutive BCL6 activity, which can have tumorigenic effects. BCL6 expression was associated with prognosis in systemic DLBCL, however with contradicting results in PCNSL (Braaten et al., 2003; Camilleri‐Broet et al., 2006; Chang et al., 2003; Momota et al., 2010; Rubenstein, Hsi, et al., 2013). Usage of different antibody clones and cutoff points for BCL6 could explain these contradicting results. Chromosomic translocation of BCL6 and Ig genes is reported in PCNSL, leading to gene overexpression associated with tumorigenesis in PCNSL (Basso & Dalla‐Favera, 2012; Cai et al., 2019; Sung et al., 2011). However, in this trial LMO2 and BCL6 expressions were not associated with the clinical outcomes.

*MGMT* and its protein are usually studied in tumorigenesis due to its potential prognostic impact and as predictor factor to alkylating agents. Its protein repairs mutagenic DNA lesion preventing mismatch and errors during DNA replication and transcription (Cai et al., 2019; Toffolatti et al., 2014). Hypermethylation of the CpG islands in the promotor region of MGMT leads to transcriptional silencing of the DNA repair enzyme O (6)‐methylguanine‐DNA methyltransferase, being reported in 37% of PCNSL (Zheng et al., 2017). Methylation has been correlated with a better overall response in patients with multiform glioblastoma and also in PCNSL (Adachi et al., 2012; Thomas et al., 2013; Zheng et al., 2017), therefore is a potential for targeted therapies. In this study, median gene expression was zero and immunohistochemistry was negative for all patients. Methylation of its promotor was not studied; however, the lack of protein expression and the low gene expression could be secondary to the methylation of its promoter (Vazquez‐Arreguin et al., 2018; Zheng et al., 2017). Yet, overexpression of *MGMT* presented tendency of worse prognosis (PFS, *p* value = 0.056), similar to literature data where genetic silencing is associated with better prognosis in patients treated with HDMTX (Cai et al., 2019; Toffolatti et al., 2014).

POU2F1 (OCT1) overexpression is associated with tumorigenesis in some studies (Vazquez‐Arreguin et al., 2018), with conflicting prognostic value described in literature: Some trials reported that overexpression of POU2F1 was associated with worse prognosis and aggressiveness in gastric, colon, and prostate cancers, among others. Some other studies did not confirm its prognostic value (Maddox et al., 2012; Vazquez‐Arreguin et al., 2018). POU2F1 is a proto‐oncogene, operating target genes associated with proliferation, immune modulation, oxidative, cytotoxic stress resistance, and metabolic regulation, among others. Further data also show that POU2F1 is involved in the high‐affinity transport of anthracyclines, and defects on OCT1 could potentially contribute to resistance of cancer cells to some chemotherapeutical agents. In a retrospective study with 77 DLBCL, POU2F1 lower expression was associated with better OS and PFS (Gouveia et al., 2020). In this trial, POU2F1 was not associated with the clinical outcomes studied.

Immune escape mechanisms have also been studied in PCNSL. PDCD1 is an immune checkpoint and protects against autoimmune responses, and the interaction of PD‐L1 on cancer cells with PD1 on T cells leads the cancer cells to escape from immune system (Menter et al., 2016). PD‐L1 has been reported in 53%–60% of tumor‐infiltrating lymphocytes (TIL) and 37% in tumoral cells in PCSNL (Berghoff et al., 2014), suggesting that immune escape may have a role in PCNSL tumorigenesis. Its prognostic value has been reported by Cho et al, who described that overexpression of PDCD1 protein, was associated with worse OS and DFS, however with different cutoff values used from this study (≥70 cells/HPF) (Cho et al., 2017). In this trial, median gene expression of PDCD1 was zero and the majority of patients (85%) did not present PDCD1 immunoexpression in tumor cell or in the TIL.

In conclusion, overexpression of genes *MYC* and *MGMT* was associated with worse outcomes in this study, implying these genes as potential biomarkers related to poor prognosis in PCNSL. Such findings need to be confirmed in future trials with a larger casuistic.

## CONFLICTS OF INTEREST

The authors declare no conflict of interest.

## AUTHOR CONTRIBUTIONS

D.G.C.R. and J.P. designed the study concept. D.G.C.R. and M.C.N.Z. performed histopathological analysis and interpretation. D.G.C.R., D.L., H.F.C., and S.P.B performed molecular experiments. D.G.C.R and D.L. handled and interpreted the data. D.G.C.R. and V.G. performed statistical analysis. D.G.C.R., L.A.P.C.L., J.P., and D.L. wrote the manuscript. J.P. and V.R. revised the manuscript. J.P. served as study coordinator.

### PEER REVIEW

The peer review history for this article is available at https://publons.com/publon/10.1002/brb3.2061.

## Data Availability

The data that support the findings of this study are available from the corresponding author upon reasonable request.
